# A Bibliometric Analysis of Clear Aligner Treatment (CAT) From 2003 to 2023

**DOI:** 10.7759/cureus.63348

**Published:** 2024-06-28

**Authors:** Xian He, Zeyu Huang, Yi Yang, Xuechun Yuan, Shangyou Wen, Yuetian Li, Guannan Hu, Wenli Lai, Hu Long

**Affiliations:** 1 State Key Laboratory of Oral Diseases & National Center for Stomatology & National Clinical Research Center for Oral Diseases & Department of Orthodontics, West China Hospital of Stomatology, Sichuan University, Chengdu, CHN; 2 West China School of Stomatology, Sichuan University, Chengdu, CHN

**Keywords:** research status, data visualization, orthodontics, clear aligner treatment, bibliometric analysis

## Abstract

Clear aligner treatment (CAT) has been evolving over the past two decades. This study aims to conduct a comprehensive and up-to-date bibliometric analysis of publications related to CAT, presenting the research trends, landscapes, and hot spots in this field.

All publications were retrieved from the Web of Science Core Collection from 2003 to 2023. In addition to a general analysis of research landscapes, the following items were analyzed, i.e., countries, institutions, authors, journals, publications, and keywords. A total of 1031 relevant publications were included in this study.

From 2003 to the present, the number of publications and citations in this field showed an increasing trend. Italy led in terms of publication counts, and Sichuan University in China had the highest publication counts among institutions. In total, 33 scholars had published a minimum of 10 articles, and the collaborations among them were mostly within each country. The American Journal of Orthodontics and Dentofacial Orthopedics published the most relevant publications. "Predictability of tooth movements," "influencing factors for clinical efficacy," "biomechanics," and "patients' perception and periodontal health" stood out as the core research focus on CAT.

Our study identified the most influential countries, institutions and authors, and their cooperative relationships, and detected hot research topics on CAT, calling for more high-quality international collaborative research in the future.

## Introduction and background

Clear aligner treatment (CAT) is an orthodontic technique that utilizes thermoplastic materials, adhesive attachments, and other auxiliary tools to move teeth incrementally through digital technologies [1-3]. Clear aligners produced by commercial companies or fabricated by clinicians in clinics or offices (often referred to as in-house aligners) have undergone three generations of development [4]. The biomechanics of clear aligners has evolved from solely relying on the aligners to incorporating attachments, complex buttons, and inter-maxillary elastics and further to utilizing digital technology for virtual design [4]. Through subtle deformations induced by dentitions, clear aligners provide more gentle and precise forces than traditional metal appliances, facilitating more predictable tooth movements. 

Compared to fixed appliance treatment (FAT), CAT has natural advantages in terms of aesthetics and comfort. Additionally, some studies indicate associations between CAT and healthier periodontal conditions, lower anxiety levels, shorter treatment duration, higher oral health-related quality of life, and significantly improved results [5-9]. Clearly, choosing CAT can result in a more pleasant orthodontic journey than FAT. 

Moreover, with developments in aligner materials and innovations in biomechanics, CAT is able to manage a variety of malocclusion and to achieve differing types of tooth movements. Some clinical studies have explored the predictability of CAT in various types of tooth movements. The results indicated that clear aligners were more efficient in achieving movements such as molar distalization and arch expansion, while the predictability was low for movements like extraction and rotation correction [3,9-12]. Clinical research on CAT still requires further refinement, and recent market growth has been driving advances in clinical studies. The CAT technique is evolving rapidly, and a growing number of scientific publications are emerging every year.

Bibliometrics is a comprehensive knowledge system that combines mathematics, statistics, and literature to quantitatively analyze and monitor published literature data, thus evaluating a discipline's progress [13]. Bibliometric analysis can be used to determine essential elements (i.e., high-impact journals, authors, and keywords), effectively assisting in research content classification and research hot spot identification [14,15].

Although a previous bibliometric analysis on CAT was published in 2021 [16], its literature searching was up to March of 2020. Since the number of publications on CAT almost doubled after 2020, a new bibliometric analysis is urgently needed to evaluate the general trends of scientific research on CAT. Moreover, the previous bibliometric analysis only focused on the most-cited 50 articles.

Therefore, we aimed to conduct an up-to-date bibliometric analysis on CAT by analyzing all pertinent scientific articles on CAT, so as to offer a comprehensive and up-to-date evaluation of research trends on CAT.

## Review

Materials and methods

Database and Search Strategy

**Figure 1:**
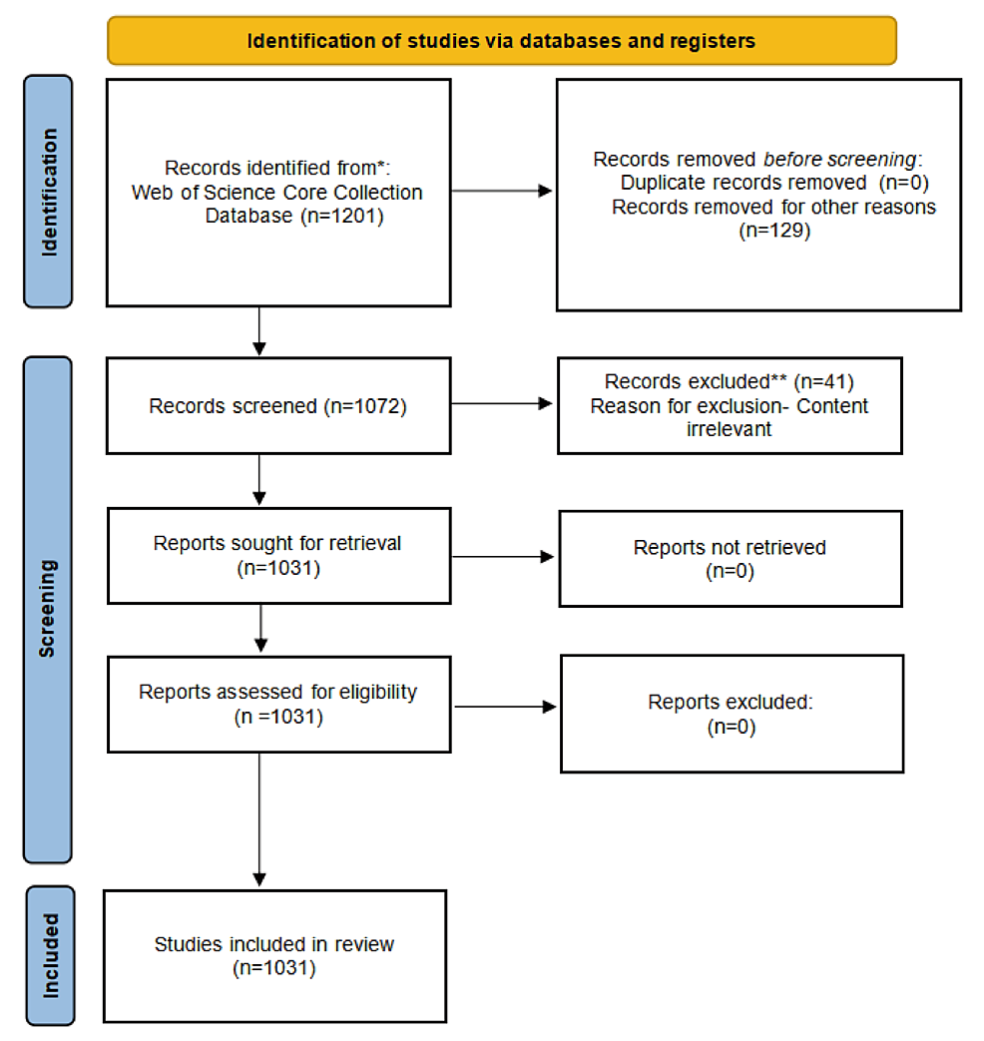
A flowchart for literature identification in the Web of Science Core Collection. Image Credit: Xian He

The literature data were sourced from the Web of Science Core Collection. To eliminate potential bias arising from database updates, all retrieval work was completed on November 30, 2023. The process of data retrieval is displayed in Figure 1.

The electronic searching was conducted by using the following search strategy: (TS=(clear OR invisible OR removable OR plastic OR orthodont OR transparent) AND TS=(aligner*)) OR TS=(Invisalign*) OR TS=(Angelalign*) OR TS=(ClearCorrect*). The searching was conducted without restrictions on publication year. Following retrieving all pertinent studies, initial screening was performed, and unrelated publications were removed after reviewing abstracts.

Scientific publications satisfying the following criteria were included in our study: (1) they were related to orthodontics and clear aligner; (2) publications in all languages were included; (3) the publication date ranged from 1997 to 2023; and (4) both basic and clinical research were included. Duplicate publications were excluded.

Data Collection and Cleaning

All literature data included were exported in two formats, "plain text file" and "tab-delimited file," encompassing both full record and cited references. The "Citation Report" of Web of Science was also collected. Specifically, the following items were included in the files: countries, institutions, authors, journals, and keywords.

Data cleaning involved eliminating duplicate publications and consolidating synonyms for the aforementioned items. The former task was accomplished using Citespace, while the latter relied on collaboration between WPS Office and VOSviewer [17].

Data Analysis and Visualization

The following analyses were conducted in this study, including research trend analysis, dual-map overlay, cooperative relationship analysis, bibliometric coupling analysis, co-occurrence analysis, and burst detection.

Specifically, research trend analysis was conducted to indicate the changes in the annual number of publications and citations. Dual-map overlay demonstrated the source of knowledge in CAT research. VOSviewer software was employed to perform cooperative analysis on collaborations among different countries, authors, and institutions. The frequency of citing the same publications between any two journals was analyzed through bibliometric coupling analysis. Co-occurrence analysis was utilized to reflect the similarity among keywords through counting the co-occurrence frequencies. Burst detection was conducted by evaluating the sudden increase in citation counts of publications and occurrence frequencies of keywords to reflect the research frontiers of CAT.

All data analysis and visualization processes were completed using WPS Office, Citespace, VOSviewer, and Pajek.

Results

General Research Trends of CAT

**Figure 2:**
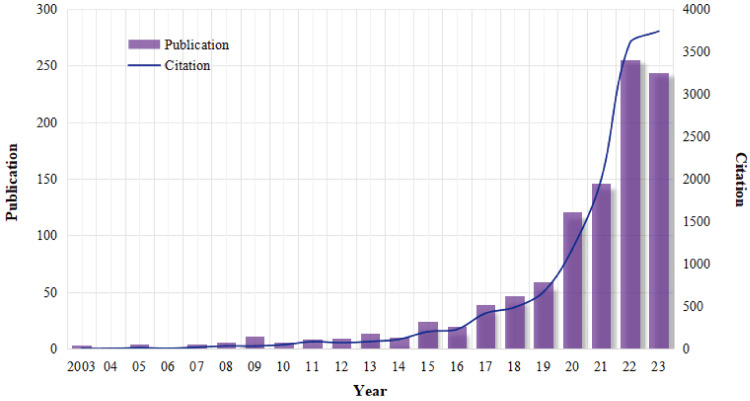
The analysis of the general research trend of CAT from 2003 to 2023. CAT: clear aligner treatment Image Credit: Xian He

A total of 1031 eligible publications (915 articles and 116 reviews) were included in this study. As displayed in Figure 2, since the first publication on CAT in 2003, the annual number of publications and citations exhibited minor fluctuations in the early years (before 2016) but demonstrated an overall upward trend for the whole time period (2003-2023). Specifically, the number of publications each year was below 50 before 2019 but increased rapidly after 2020. Moreover, the number of publications was around 150 in 2021, which was almost doubled in 2022 and 2023. Similar trends were found for the citations on CAT.

**Figure 3:**
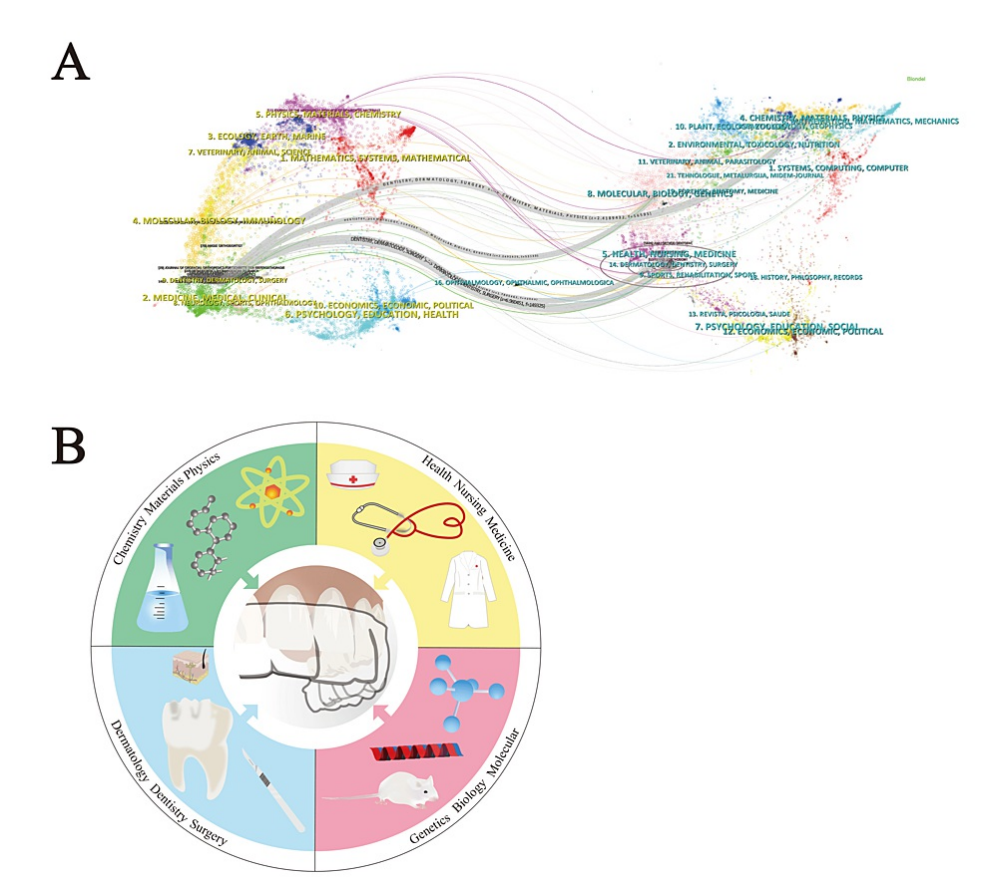
(A) A dual-map overlay of CAT and (B) the knowledge flow process of "dentistry, dermatology, and surgery." The right side of the dual-map overlay displays the disciplines to which the knowledge sources of CAT belong, while the left side shows the disciplines of knowledge distribution in the CAT field. The lines between them indicate the process of knowledge flowing from the right side to the left side. "Dermatology, dentistry, and surgery," "Health, nursing, and medicine," "Chemistry, materials, and physics," and "Genetics, biology, and molecular" are the main knowledge sources of CAT. CAT: clear aligner treatment Image Credit: Xian He

The dual-map overlay showed that the majority of publications on CAT cited the publications from the following four disciplines: "Dermatology, dentistry, and surgery," "Health, nursing, and medicine," "Chemistry, materials, and physics," and "Genetics, biology, and molecular" (Figure 3A, 3B).

Analysis of Cooperative Relationship

**Figure 4:**
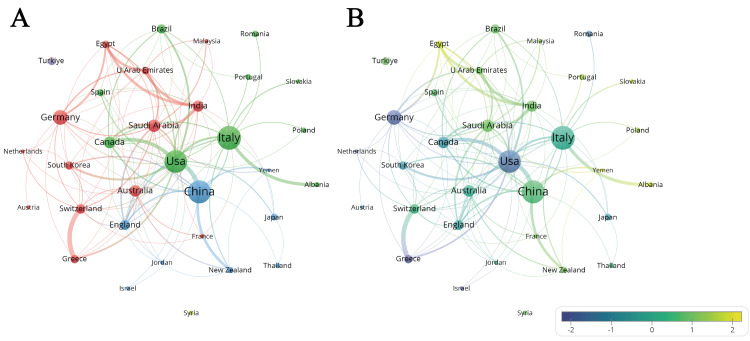
(A) A network visualization of countries and (B) an overlay visualization of countries with normalized timeline. Circular nodes represent the research objects, and the node size is determined collectively by collaboration quantity and strength, publication counts, and citation counts. The lines between nodes represent collaborative relationships, with the size determined by the strength of collaboration. In network visualization, objects belonging to the same cluster are assigned the same color. In overlay visualization, node color is determined by the average publication year of the node, and after normalization, blue represents articles published earlier on average, while yellow represents articles published later on average. Image Credit: Xian He and Zeyu Huang

Countries: A total of 67 countries participated and published papers in the field of CAT, with 33 countries publishing at least five publications. The top 10 countries in terms of publication counts are displayed in Table 1. Among them, Italy (n=205, 19.88%), China (n=196, 19.01%), and the United States (n=185, 17.94%) were the top three countries. In addition, the United States exhibited the most citations and average citations (3392, 18.34). Through VOSviewer's co-authorship analysis of countries, the 33 countries can be categorized into three main clusters (Figure 4A). The green cluster was centered around the United States and Italy, the blue cluster was centered around China, and the red cluster exhibited a multi-centered pattern involving countries like Germany and Saudi Arabia. These central countries were particularly active in their respective collaboration domains, leading the research related to CAT within their clusters. The United States was the country with the most extensive collaborations with other nations, exhibiting close collaborations with China, Canada, and South Korea. As displayed in Figure 4B, research on CAT was initiated by the United States and Germany, while China and Italy gradually caught up with the aforementioned countries recently.

**Table 1:** Top 10 countries with the most numbers of publications in CAT. ^1^We regard authors who simultaneously meet two criteria, (1) being the top two productive authors in their countries and (2) publishing at least five documents, as individuals making leading contributions to CAT in that country. CAT: clear aligner treatment

Rank	Country	Documents	Citations	Average citations	Leading contributions^1^
1	Italy	205	2984	14.56	Castroflorio, Tommaso (25); Lombardo, Luca (25)
2	China	196	1773	9.05	Lai, Wenli (13); Long, Hu (12)
3	United States	185	3392	18.34	Huang, Greg (13); Lindauer, Steven J. (11)
4	Germany	78	1421	18.22	Bourauel, Christoph (11); Keilig, Ludger (10)
5	Saudi Arabia	56	264	4.71	Baeshen, Hosam Ali (5)
6	Canada	50	755	15.10	Flores-Mir, Carlos (10); El-bialy, Tarek (8)
7	Australia	50	481	9.62	Weir, Tony (22); Freer, Elissa (11)
8	India	45	273	6.07	Vaid, Nikhilesh (19)
9	England	37	393	10.62	Fleming, Padhraig S. (5)
10	Switzerland	35	568	16.23	Eliades, Theodore (22); Koletsi, Despina (7)

**Figure 5:**
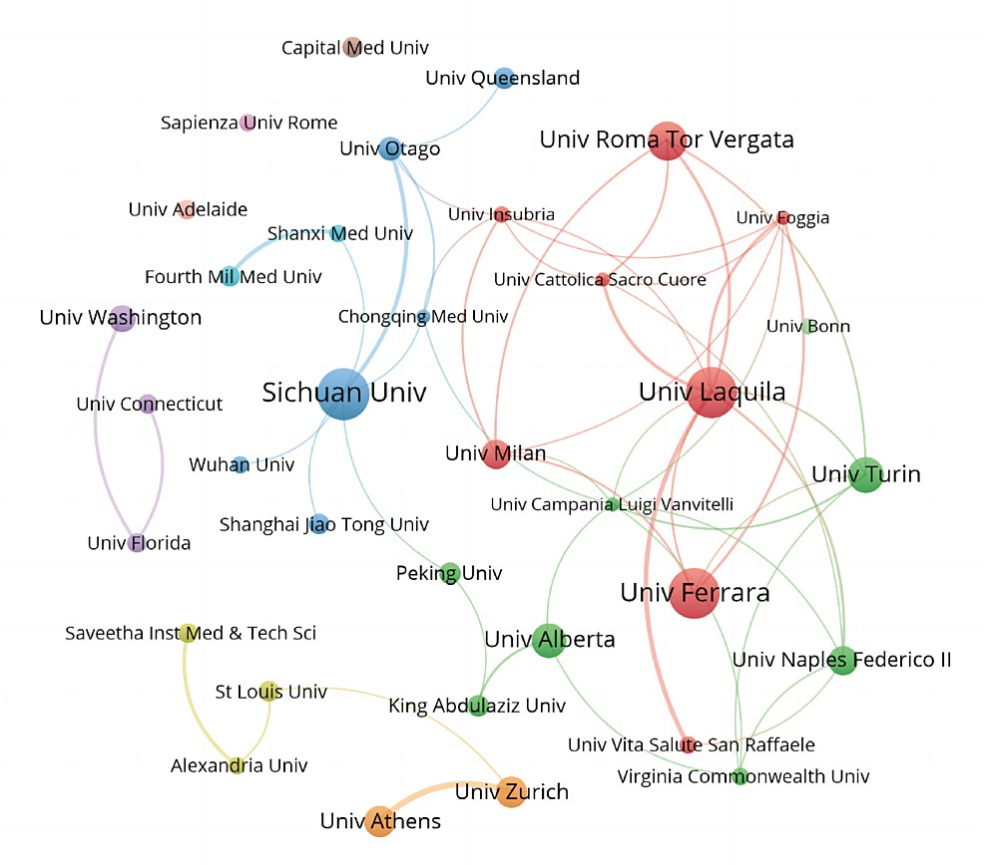
The cooperative relationship of the top 35 institutions. Image Credit: Xian He and Zeyu Huang

Institutions: A total of 900 institutions participated in CAT-related research and published relevant articles, with 35 institutions having published at least 10 papers (Figure 5). Considering both publication counts and collaboration strength, Sichuan University and the University of L'Aquila are the most prominent ones, leading the development of the CAT discipline within their respective clusters. As displayed in Table 2, in terms of publication counts, the top 10 institutions are listed, with Sichuan University (n=35), the University of Ferrara (n=34), and the University of L'Aquila (n=34) being the top three institutions in this field. In terms of citation counts, the University of Turin (n=779) from Italy was the most influential unit in this domain.

**Table 2:** Top 10 institutions with the most number of publications in CAT. CAT: clear aligner treatment

Rank	Institution	Documents	Citations	Average citations	Average publication year	Countries
1	Sichuan University	35	272	7.77	2021	China
2	University of Ferrara	34	432	12.71	2020	Italy
3	University of L'Aquila	34	302	8.88	2021	Italy
4	University of Rome Tor Vergata	26	181	6.96	2022	Italy
5	University of Turin	24	779	32.46	2020	Italy
6	University of Alberta	23	431	18.74	2020	Canada
7	National and Kapodistrian University of Athens	21	461	21.95	2019	Greece
8	University of Zurich	21	392	18.67	2020	Switzerland
9	University of Naples Federico II	20	607	30.35	2021	Italy
10	University of Milan	20	247	12.35	2021	Italy

**Figure 6:**
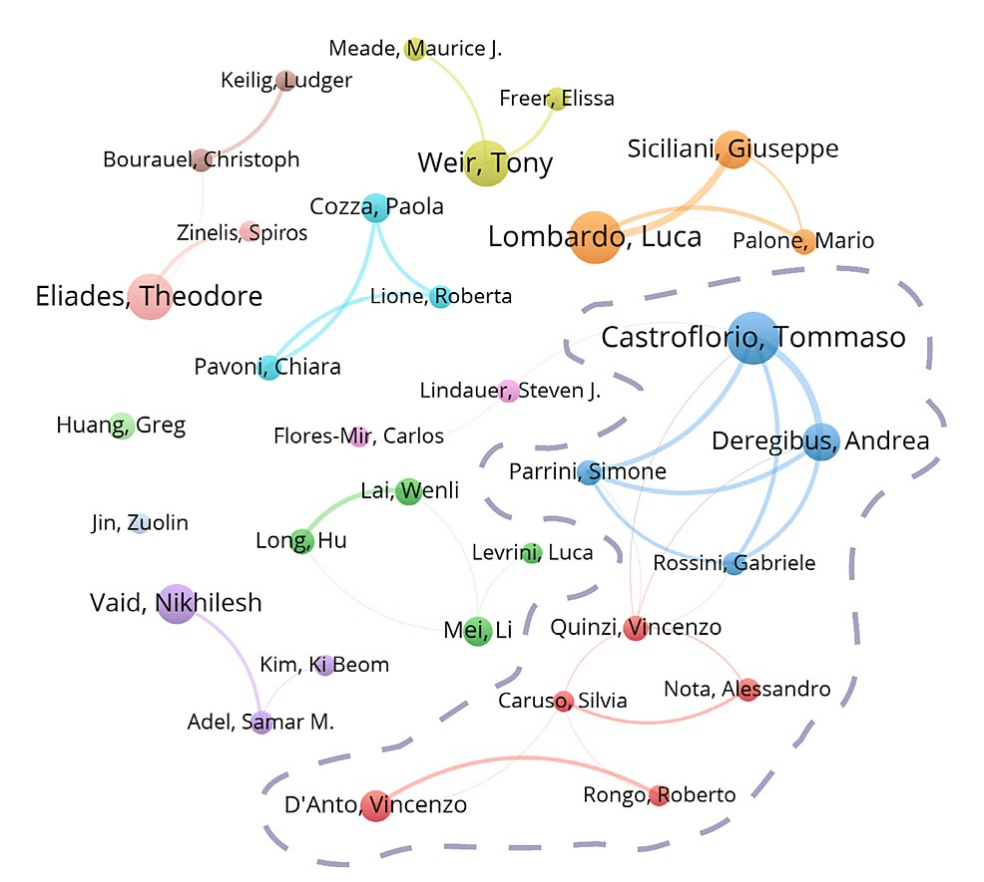
The cooperative relationship of the top 33 authors. The dashed box shows the cooperative network formed by Italian scholars. Image Credit: Xian He and Zeyu Huang

Authors: A total of 3407 authors participated in the CAT-related research, with 33 authors having published at least 10 publications (Table 3). Among them, Tommaso Castroflorio from the University of Turin and Luca Lombardo from the University of Ferrara published 25 articles, marking their substantial contributions to the field. In terms of citations, Tommaso Castroflorio (n=792), Andrea Deregibus (n=700), and Theodore Eliades (n=667) ranked as the top three authors. Moreover, the average publication years for these 33 leading scholars were after 2017. As shown in Figure 6, the majority of the collaborations was intra-country cooperation, with limited international collaboration. Of particular, in intra-country collaborations, Italian scholars, led by Tommaso Castroflorio, formed the largest collaborative unit in the visualization map.

**Table 3:** Productive authors with at least 10 publications on CAT. CAT: clear aligner treatment

Rank	Author	Documents	Citations	Average publication year	Average citations	Affiliated institution	Country
1	Castroflorio, Tommaso	25	792	2019	31.68	University of Turin	Italy
2	Lombardo, Luca	25	371	2020	14.84	University of Ferrara	Italy
3	Eliades, Theodore	22	667	2018	30.32	University of Zurich	Switzerland
4	Weir, Tony	22	83	2022	3.77	University of Adelaide	Australia
5	Vaid, Nikhilesh	19	353	2021	18.58	Saveetha Institute of Medical and Technical Science	India
6	Deregibus, Andrea	18	700	2019	38.89	University of Turin	Italy
7	Siciliani, Giuseppe	18	360	2019	20.00	University of Ferrara	Italy
8	D'Anto, Vincenzo	15	196	2021	13.07	University of Naples Federico II	Italy
9	Cozza, Paola	14	91	2022	6.50	Saint Camillus International University of Health and Medical Sciences	Italy
10	Mei, Li	14	110	2021	7.86	University of Otago	New Zealand
11	Huang, Greg	13	326	2016	25.08	University of Washington	United States
12	Lai, Wenli	13	121	2021	9.31	Sichuan University	China
13	Long, Hu	12	105	2022	8.75	Sichuan University	China
14	Palone, Mario	12	73	2022	6.08	University of Ferrara	Italy
15	Parrini, Simone	12	536	2020	44.67	University of Turin	Italy
16	Pavoni, Chiara	12	47	2022	3.92	University of Rome Tor Vergata	Italy
17	Quinzi, Vincenzo	12	148	2021	12.33	University of L'Aquila	Italy
18	Adel, Samar M.	11	61	2022	5.55	Alexandria University	Egypt
19	Bourauel, Christoph	11	508	2019	46.18	University of Bonn	Germany
20	Freer, Elissa	11	56	2021	5.09	University of Queensland	Australia
21	Lindauer, Steven J.	11	176	2021	16.00	Virginia Commonwealth University	United States
22	Lione, Roberta	11	46	2022	4.18	Saint Camillus International University of Health and Medical Sciences	Italy
23	Meade, Maurice J.	11	49	2022	4.45	University of Adelaide	Australia
24	Nota, Alessandro	11	195	2020	17.73	Vita-Salute San Raffaele University	Italy
25	Rossini, Gabriele	11	558	2019	50.73	University of Turin	Italy
26	Caruso, Silvia	10	165	2020	16.50	University of L'Aquila	Italy
27	Flores-Mir, Carlos	10	386	2018	38.60	University of Alberta	Canada
28	Jin, Zuolin	10	24	2023	2.40	Fourth Military Medical University	China
29	Keilig, Ludger	10	339	2020	33.90	University of Bonn	Germany
30	Kim, Ki Beom	10	150	2022	15.00	Saint Louis University	United States
31	Levrini, Luca	10	173	2019	17.30	University of Insubria	Italy
32	Rongo, Roberto	10	127	2021	12.70	University of Naples Federico II	Italy
33	Zinelis, Spiros	10	240	2018	24.00	National and Kapodistrian University of Athens	Greece

Analysis of Journals

**Figure 7:**
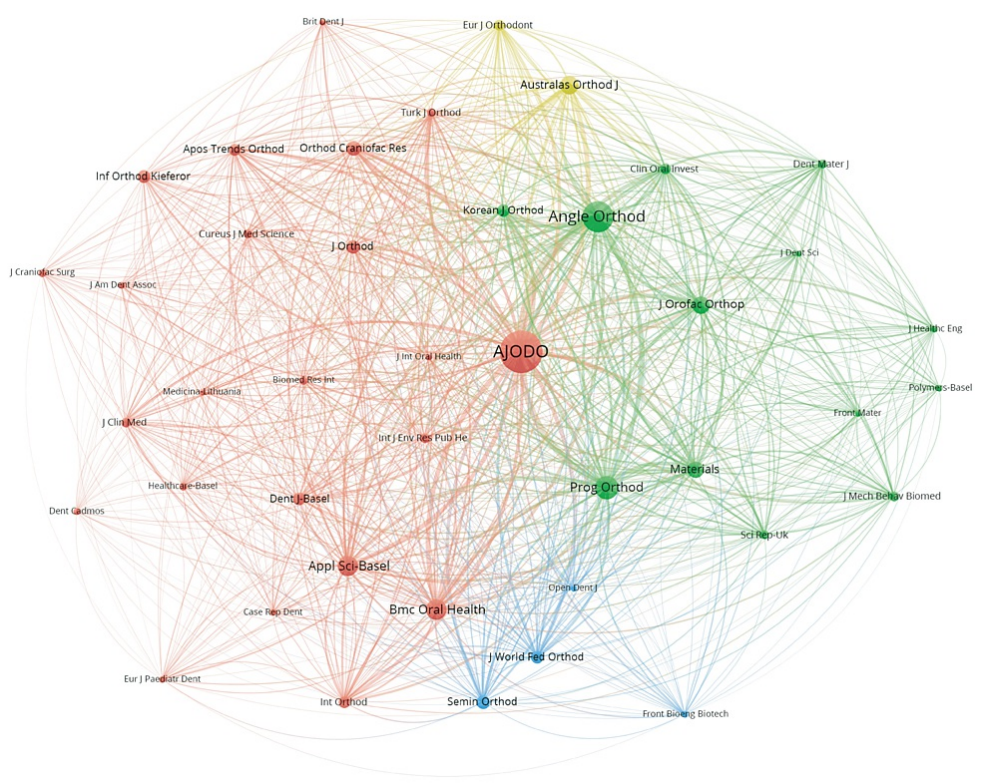
The bibliographic coupling analysis of journals. In this graph, nodes represent journals, and their size is determined by a combination of publication counts, citation counts, and the numbers and strength of links. The lines connecting nodes represent bibliographic coupling relationships between journals. Image Credit: Xian He and Zeyu Huang

A total of 199 journals (Australian Orthodontic Journal was renamed the Australasian Orthodontic Journal in 2017 and the two are considered as the same journal) had published articles in the field of CAT, with 23 journals having published at least 10 articles in this domain. The top 10 journals with the most publication outputs are listed in Table 4. Specifically, the top 10 journals collectively published 470 articles, accounting for 45.59% of the total. The American Journal of Orthodontics and Dentofacial Orthopedics (AJODO) (133 publications, 2863 citations), The Angle Orthodontist (79 publications, 2351 citations), and Progress in Orthodontics (47 publications, 898 citations) were recognized as authoritative journals in the orthodontic field, ranking the top three in both publication counts and citation counts. As displayed in Figure 7, AJODO, The Angle Orthodontist, and Progress in Orthodontics exhibited high bibliographic coupling frequency, indicating a significant degree of similarity in the CAT-related research published by these three journals.

**Table 4:** Top 10 journals publishing the most number of publications in CAT. CAT: clear aligner treatment

Rank	Journal	Documents	Citations	Average publication year	Average citations	IF (2022)
1	American Journal of Orthodontics and Dentofacial Orthopedics	133	2863	2019	21.53	3.0
2	The Angle Orthodontist	79	2351	2018	29.76	3.4
3	Progress in Orthodontics	47	898	2020	19.11	4.8
4	BMC Oral Health	40	814	2021	20.35	2.9
5	Applied Sciences-Basel	39	80	2022	2.05	2.7
6	Australasian Orthodontic Journal (Australian Orthodontic Journal)	32	149	2019	4.66	0.4
7	Journal of Orofacial Orthopedics	29	502	2019	17.31	1.7
8	Materials	28	295	2021	10.54	3.4
9	Orthodontics & Craniofacial Research	22	392	2021	17.82	3.1
10	Seminars in Orthodontics	21	146	2020	6.95	4.2

Burst Detection of Publications

**Figure 8:**
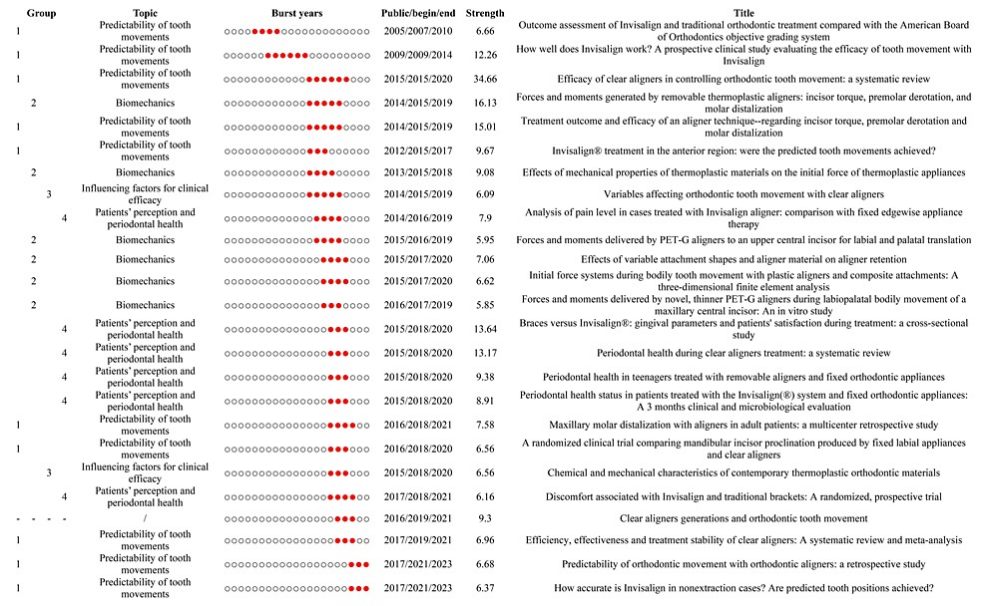
The burst detection of the top 25 publications. Based on the research content, 25 burst publications can be broadly categorized into the following four topics: predictability of tooth movements, influencing factors for clinical efficacy, biomechanics, and patients' perception and periodontal health. Each circle in the "burst years" column represents a single year. The red-marked circle signifies that within the respective year, the publication corresponding to that row exhibited a burst phenomenon. Image Credit: Xian He

**Figure 9:**
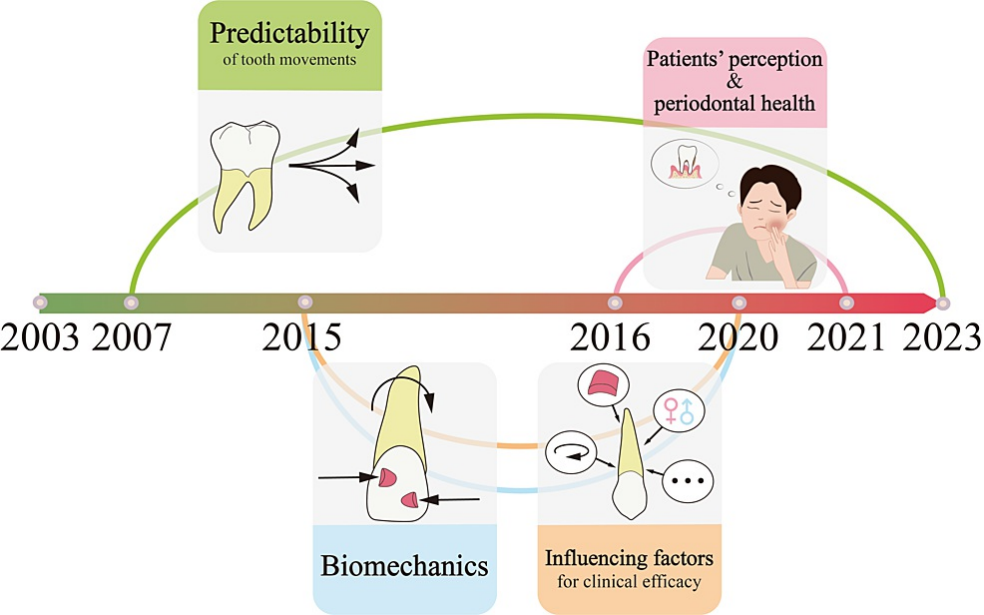
The time span of the four research topics. The burst years of "predictability of tooth movements," "biomechanics," "influencing factors for clinical efficacy," and "patients' perception and periodontal health" were 2007-2023, 2015-2020, 2015-2020, and 2016-2021, respectively. Image Credit: Xian He

The 25 strongest burst publications are displayed in Figure 8. Based on the contents of their research, we categorized 24 articles (excluding a review article) into four groups. Group 1, group 2, group 3, and group 4 corresponded to the research topics "predictability of tooth movements," "biomechanics," "influencing factors for clinical efficacy," and "patients' perception and periodontal health," respectively. As shown in Figure 9, "predictability of tooth movements" was a persistent topic since its emergence in 2007, while "biomechanics" gained considerable attention from 2015 to 2020. Moreover, the other two topics ("influencing factors for clinical efficacy" and "patients' perception and periodontal health") were focused between 2015 and 2020 and between 2016 and 2021, respectively.

Analysis of Keywords

**Figure 10:**
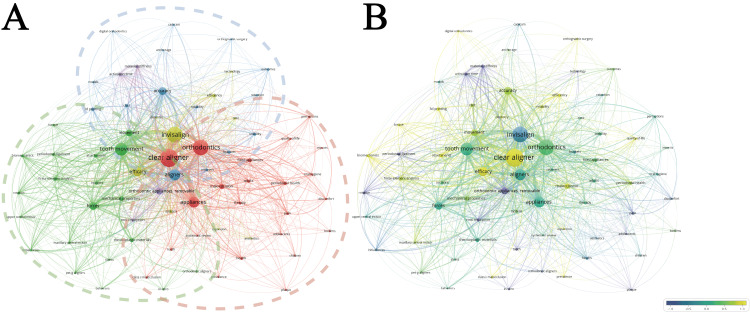
(A) A network visualization of co-occurrence keywords and (B) an overlay visualization of keywords with normalized timeline. The size of nodes in the co-occurrence analysis graph is determined by occurrence times, co-occurrence times, and the co-occurrence strength of keywords. The size of lines between nodes is determined by the co-occurrence strength of the keywords. Image Credit: Xian He and Zeyu Huang

A total of 2669 author keywords and keywords plus were extracted. In Figure 10A, 71 keywords that appeared more than 15 times were included in the analysis, and these keywords were categorized into five clusters (red, green, blue, yellow, purple) in the network visualization. There were three clusters (red, green, blue) containing more than 10 keywords. Specifically, the red cluster was related to the subjective feelings of treatment and oral health conditions of patients, including typical keywords such as quality of life, perceptions, pain, periodontal health, and oral hygiene. The green cluster pertained to the biomechanics of tooth movements, including typical keywords such as tooth movement, forces, mechanical properties, finite element analysis, and biomechanics. The blue cluster focused on CAT-related technologies, encompassing typical keywords like CBCT, 3D printing, CAD/CAM, digital orthodontics, and anchorage. The average occurrence time of the aforementioned keywords is shown in Figure 10B. Keywords such as activation time, material stiffness, and root resorption appeared earlier on average. In contrast, keywords like digital orthodontics, 3D printing, and quality of life emerged later on average. Meanwhile, keywords such as Invisalign, tooth movement, and forces, which were frequently presented in early research, have maintained a long-lasting attention by researchers.

**Figure 11:**
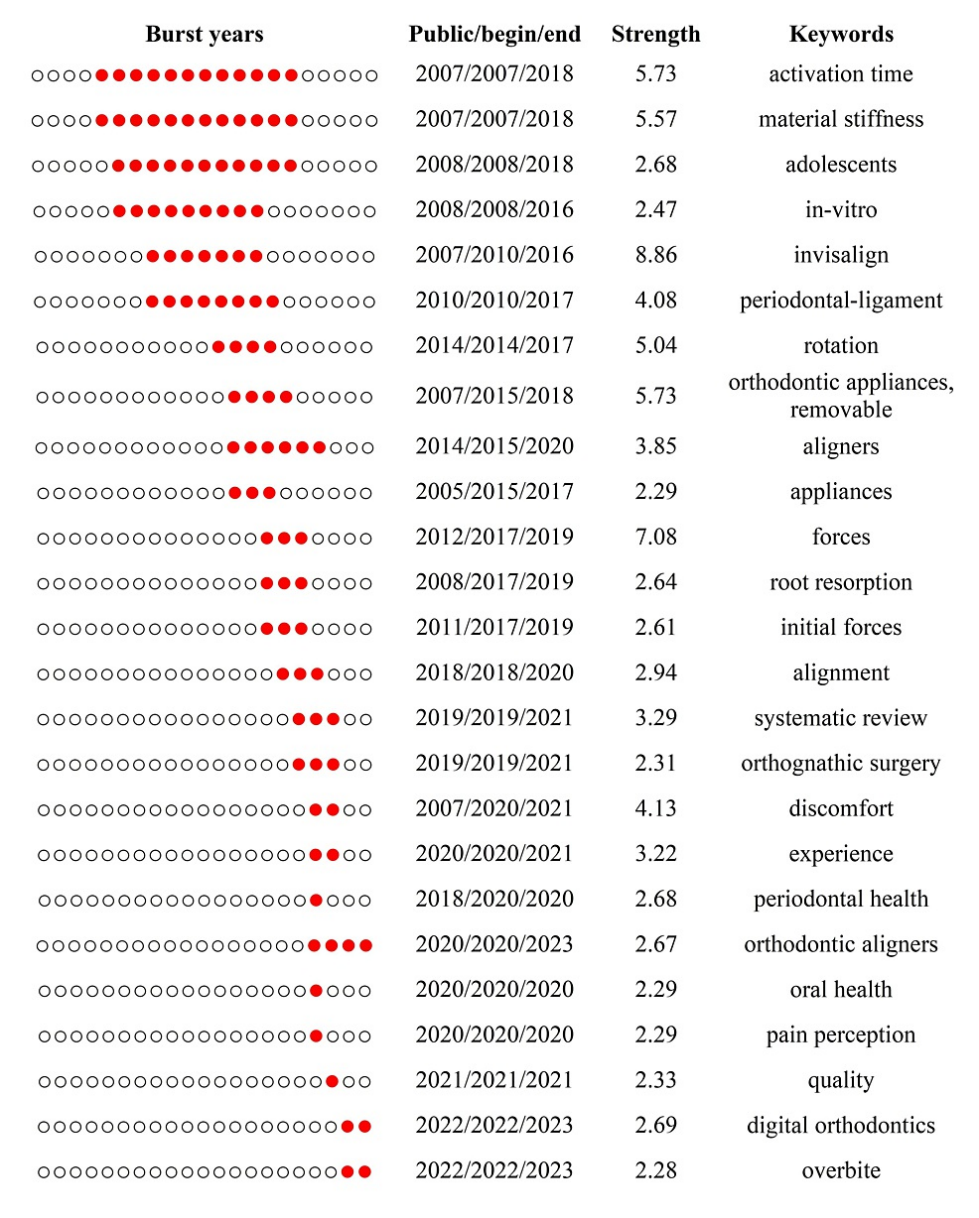
The burst detection of the top 25 keywords. Image Credit: Xian He

The 25 strongest burst keywords are displayed in Figure 11. In the early stages of CAT research, particularly before 2010, bursts were mainly associated with the properties of materials, the basic structure of the tooth, and periodontal tissues, giving rise to keywords such as activation time, material stiffness, and periodontal ligament. Between 2010 and 2017, bursts were predominantly linked to tooth movements and biomechanics, introducing keywords like rotation, forces, and initial forces. From 2017 to the present, keywords were more closely related to subjective experiences and objective oral health conditions, as well as technological innovations. Representative burst keywords in this period included discomfort, oral health, and digital orthodontics.

Discussion

We conducted a bibliometric analysis of 1031 CAT-related publications between 2003 and 2023 and analyzed countries, institutions, authors, journals, publications, and keywords to present the research status of CAT.

The annual number of publications in the field of CAT showed a steady growing trend before 2019 with an overall low pace. The potential reason is that many publications before 2019 discussed the low efficacy of clear aligners [18] and the undesirable properties of early-stage CAT materials [19], discouraging practitioners from using clear aligners in clinical practice. Corresponding to these concerns, the rudimentary form of clear aligner was only suitable for treating simple cases, e.g., mild to moderate crowding, while not effective in managing severe malocclusion [4]. In some specific clinical scenarios, the disadvantages of CAT compared to FAT were evident [7]. The biomechanical limitations of CAT and material deficiencies undoubtedly constituted the most fundamental reasons limiting its clinical application.

With the introduction of SmartTrack materials by Invisalign company in 2013 and the expiration of some core techniques that were patented by Invisalign in 2017, the emergence of more reliable treatment protocols and an increasing number of industry competitors injected new vitality into CAT. Moreover, due to the outbreak of the COVID-19 pandemic, the advantages of CAT over FAT were realized [20]. Practitioners who were compelled to work from home had more time to focus on the outputs of clinical research. This may explain the sudden surge of publications on CAT in 2020.

The dual-map overlay revealed that "Dermatology, dentistry, and surgery," "Health, nursing, and medicine," "Chemistry, materials, and physics," and "Genetics, biology, and molecular" were the most crucial sources of knowledge in the field of CAT. Current research related to clear aligners encompass a variety of topics, ranging from clinical applications in dentistry, patients' subjective feelings, materials and biomechanics, and periodontal health and microorganisms. These topics are consistent with those in the aforementioned four categories, which may explain the knowledge flow directions.

Italy, China, and the United States stood out as the top three countries making the most substantial contributions to the CAT field. The United States, being the birthplace of CAT, naturally took the lead in initiating CAT research from its inception [21-23]. Greg Huang from the University of Washington and Steven Lindauer (the Editor-in-Chief of The Angle Orthodontist) have made significant contributions to the development of CAT in the United States. After the Food and Drug Administration approved Align Technology's use of Invisalign for orthodontic treatment in 1998, CAT began to spread globally, and it reached European countries (e.g., Italy) in 2001 [1,2]. The outstanding intra-country research collaborations and numerous leading scholars have led to remarkable achievements for Italy in the field of CAT. Specifically, six institutions from Italy were ranked among the top 10 institutions globally, and 16 authors from Italy were within the top 33, in which Tommaso Castroflorio from the University of Turin was recognized as the most influential scholar. Benefiting from a vast market due to its enormous population base, China had significant research achievements despite initiating CAT research relatively late (2011). Their leading scholars Wenli Lai and Hu Long from the top dental school and hospital (West China Hospital of Stomatology, Sichuan University) in China have been leading the research and clinical innovation on CAT.

AJODO published the most CAT-related publications and obtained the most citations, while The Angle Orthodontist obtained the most average citations (29.76). Consistently, these two journals were the top two journals in a previous bibliometric study due to the fact that both journals had published 14 out of 50 highly cited publications [16]. Although Progress in Orthodontics initiated relatively late in publishing articles on CAT (with an average publication year of 2020), numerous articles were being published recently.

The increasing number and decreasing strength of burst publications and keywords in recent years may suggest that CAT has entered a phase of rapid and dynamic development in research hot spots. We summarized four research hot spots in CAT: "predictability of tooth movements," "biomechanics," "influencing factors for clinical efficacy," and "patients' perception and periodontal health."

The "predictability of tooth movements" was the earliest topic of concern in CAT. Its emergence reflected clinical practitioners' lack of confidence in the early-stage application of clear aligners. Early research on this topic did reveal limitations of CAT in certain specific tooth movements [18,24]. However, with advancements in materials and techniques, the capability of CAT has been progressively enhanced and gained wider acceptance by practitioners [25,26]. Being a milestone publication in the CAT field, a systematic review published in 2015 by Rossini et al. [3] elucidated the efficiency of tooth movements in CAT, outlining both the advantages and disadvantages of clinical applications. This article sparked researchers' enthusiasm for more comprehensive studies on the "predictability of tooth movements," further refining the performance of CAT in this topic [27-31]. To some extent, it aroused scholars' attention to the topics of "biomechanics" (from 2015 to 2020) and "factors influencing clinical efficacy" (from 2015 to 2020).

Early studies on "biomechanics" were mostly based on physical models of biomechanical analysis systems [19,32-35], while three-dimensional finite-element analysis has become the mainstream approach for biomechanical analysis in CAT recently [36]. During the same period when "biomechanics" received widespread attention, another research topic, "influencing factors for clinical efficacy," was also gaining attention, probably due to its potential to enhance tooth movement efficacy. Studies on this topic have confirmed that certain patient factors (age, gender) [37,38] and treatment factors (frequency of aligner changes, types of aligner materials, and attachment positions and their shapes) [39-43] were able to affect the efficiency of tooth movements. However, since limited clinical subjects were available for the study on the influencing factors for clinical efficacy at that time period as compared to the other three topics (e.g., physical models rather than clinical subjects were needed for biomechanical research), fewer burst publications were detected on this specific topic.

With the development in aligner materials and techniques, the clinical efficacy of CAT has been gradually improved, encouraging scholars to explore more sophisticated aspects of CAT beyond its clinical efficacy. Therefore, patients' subjective feelings and periodontal health related to CAT gained widespread attention from 2016 to 2021. Specifically, numerous clinical studies were conducted on this specific topic and revealed that higher ratings of subjective feelings were reported among patients receiving CAT than among those receiving fixed appliances [6,44-46]. Meanwhile, CAT was more advantageous than FAT in maintaining periodontal health during orthodontic treatment [46-48]. Interestingly, the burst publications in the field of CAT shifted back to the topic of "predictability of tooth movements" after 2021, indicating more and more practitioners and researchers are focusing on this specific topic recently. Keywords are reflecting the research contents of CAT. The co-occurrence analysis showed that "the subjective feelings of treatment and oral health conditions of patients," "biomechanics of tooth movements," and "CAT-related technologies" are the most widely distributed clusters of keywords in the CAT field. The burst keywords reflected the transition of CAT-related research content from "the properties of materials, the basic structure of the tooth, and periodontal tissues" to "tooth movements and biomechanics" and further to "subjective experiences and objective oral health conditions, as well as technological innovations." These results were consistent with the findings in burst publications, demonstrating the driving role of technological innovations (such as materials, CBCT, digital orthodontics, 3D printing, etc.) in the development of the CAT field.

Two potential sources of bias in this study may include exclusive selection of Web of Science Core Collection as the primary data source and no inclusion of publications after the retrieval process.

## Conclusions

The past 20 years have witnessed a significant increase in the number of publications and citations related to CAT. Italy, China, and the United States are the top three countries contributing to CAT research. Sichuan University has the highest publication output, while the University of Turin exhibits the highest citation count. Research collaborations among different authors are mostly within each country, calling for more international cooperation. AJODO has the highest publication output and citation count, while The Angle Orthodontist presents the highest average citations. "Predictability of tooth movement," "influencing factors for clinical efficacy," "biomechanics," and "patients' perception and periodontal health" are hot research topics related to CAT.
